# RNA-Seq vs Dual- and Single-Channel Microarray Data: Sensitivity Analysis for Differential Expression and Clustering

**DOI:** 10.1371/journal.pone.0050986

**Published:** 2012-12-10

**Authors:** Alina Sîrbu, Gráinne Kerr, Martin Crane, Heather J. Ruskin

**Affiliations:** 1 Centre for Scientific Computing and Complex Systems Modelling, Dublin City University, Dublin, Ireland; 2 Institute for Scientific Interchange Foundation, Turin, Italy; 3 Signaling and Functional Genomics, German Cancer Research Center, Heidelberg, Germany; University College London, United Kingdom

## Abstract

With the fast development of high-throughput sequencing technologies, a new generation of genome-wide gene expression measurements is under way. This is based on mRNA sequencing (RNA-seq), which complements the already mature technology of microarrays, and is expected to overcome some of the latter’s disadvantages. These RNA-seq data pose new challenges, however, as strengths and weaknesses have yet to be fully identified. Ideally, Next (or Second) Generation Sequencing measures can be integrated for more comprehensive gene expression investigation to facilitate analysis of whole regulatory networks. At present, however, the nature of these data is not very well understood. In this paper we study three alternative gene expression time series datasets for the *Drosophila melanogaster* embryo development, in order to compare three measurement techniques: RNA-seq, single-channel and dual-channel microarrays. The aim is to study the state of the art for the three technologies, with a view of assessing overlapping features, data compatibility and integration potential, in the context of time series measurements. This involves using established tools for each of the three different technologies, and technical and biological replicates (for RNA-seq and microarrays, respectively), due to the limited availability of biological RNA-seq replicates for time series data. The approach consists of a sensitivity analysis for differential expression and clustering. In general, the RNA-seq dataset displayed highest sensitivity to differential expression. The single-channel data performed similarly for the differentially expressed genes common to gene sets considered. Cluster analysis was used to identify different features of the gene space for the three datasets, with higher similarities found for the RNA-seq and single-channel microarray dataset.

## Introduction

Analysis of the gene expression process has been an important topic for many years [1], as it can have outcomes important for understanding the way in which genetic information is processed, as well as the mechanisms involved in both natural and abnormal processes. With the developments of microarray technologies, which allow for gene expression quantification for a very large number of genes at the same time, this analysis has moved from gene to genome level [1]. Several pre-processing and analysis tools such as machine learning and reverse engineering algorithms have been tailored specifically for these data (e.g. [2], [3] and references therein). This, together with relatively low cost, has facilitated wide usage of microarrays over the past years [4]. However, some challenges still persist in working with these data, where these are related to noise introduced at different experimental and analysis stages, and/or limitation of probes to known genes [5].

**Table 1:** Gene expression datasets for *Drosophila melanogaster* embryo development.

Dataset	Number of time points	Number of replicates	Sampling interval	Hours after egg laying
NGS	12	3–4 (technical)	2 h	2–24 h
DC	7	3 (biological)	1–3 h	2 h, 3 h, 6–10 h
SC	12	3 (biological)	1 h	1–12 h

Recent advances in high throughput sequencing technologies (Next or Second Generation Sequencing) have introduced a new alternative to microarrays, namely RNA-seq [4]. This quantifies gene expression by sequencing short strands of cDNA, aligning sequences obtained back to the genome or transcriptome, and counting the aligned reads for each gene. This technology is expected to overcome some of the disadvantages of microarrays. For instance, it is able to identify transcripts that have not been previously annotated [5] and it can quantify both very low transcripts (unlike microarrays where there is background noise interference) [4], and very high ones (where microarrays suffer from *hybridisation saturation*, i.e. only a limited amount of cDNA can hybridise to a microarray spot) [5]. At the moment, although significant efforts have been made to modify algorithms and technologies, problems still exist with obtaining quantified transcription data. Some of these relate to read errors, short read mapping, SNPs, RNA splicing and sequencing depth, which particularly affect analysis of more complex transcriptomes [4]. Additionally, the experimental cost for these technologies is still very high compared to microarrays [5]. However, improvements are expected as the length of reads is increased [5] and new algorithms and methods are developed, so that RNA-seq will eventually become a more accessible tool for gene expression analysis.

Meanwhile, the objective must be to understand the nature of these data, and what they add, or hope to add, to the gene expression picture [6]. Efforts have been made to analyse compatibility and complementarity of datasets with respect to general expression patterns [4], [7], splice junctions [8], [9], and differential expression [10]. Results from these studies show good correlation between microarray (including exon arrays) and RNA-seq expression levels (reported Spearman rank and Pearson correlation values between 0.55 and 0.85 [8], [11], [12]). However, RNA-seq experiments were shown to be more suitable than microarrays for quantifying absolute gene expression levels, when validated with mass spectrometry measurements [7]. RNA-seq data have been found to display more sensitivity to differential expression tests compared to microarrays, with the number of identified genes generally larger [13], [14]. Additionally, the new platform was shown to display better discrimination of differentially expressed genes with very large expression values, while microarrays were reported better for very low transcript concentrations [10], [14], [15] (a somewhat surprising fact given that NGS data have been postulated to have an advantage for low transcript quantification). For sample classification it has been shown [16] that no significant difference between Agilent and Illumina technologies exists.

**Figure 1:**
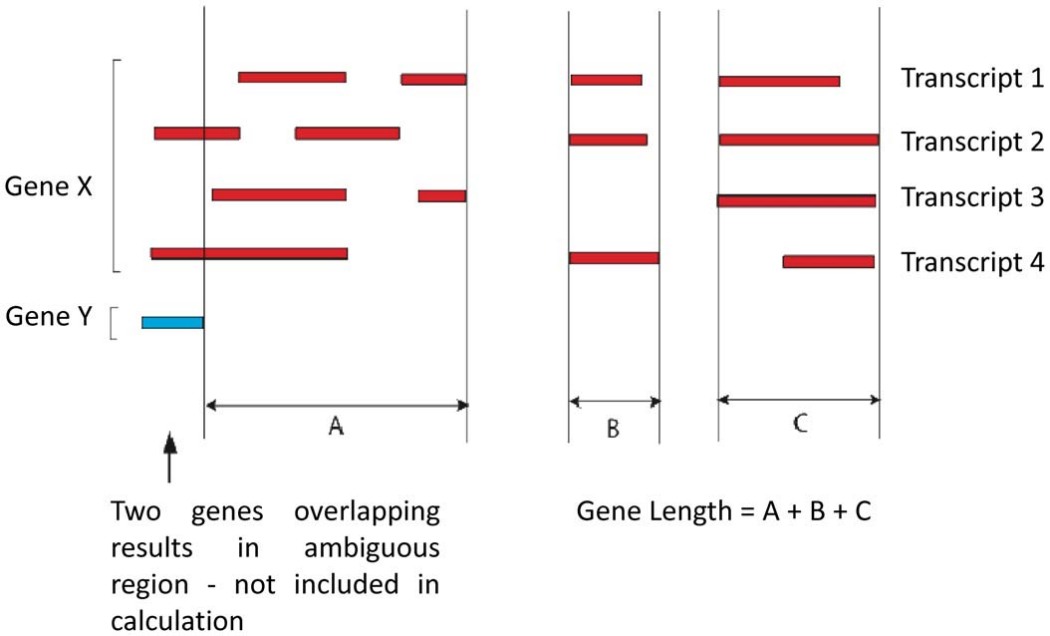
Calculation of gene length.

These studies mostly concentrate on the same samples measured with the different technologies, in order to eliminate biases due to biological variability, which allows for a more robust test of advantages and disadvantages of each platform. However, in the context of large-scale integration, more heterogeneous datasets, from different sources and samples, should also be analysed and overlapping features identified in the more general setting. Even if samples are different, it is expected that, if they measure the same process, they should underline the same overall features (e.g. differentially expressed (DE) genes, clusters). In consequence, a discussion of overlapping features in a broader setting, which may allow further integration of these data, is presented here. A detailed analysis of the gene space structure (i.e. clustering) is needed, which has not yet been performed to our knowledge. This is important as, in principle, the space structure should be similar for different technologies, since genes involved in similar processes cluster together, regardless of what measurement technology is used. However, each technology has its own characteristics, which may interfere with the clusters formed. A study of the overall space can help to identify both specific and common features for each dataset. For this, three gene expression time series datasets measuring embryo development for *Drosophila melanogaster* (RNA-seq (NGS - Next Generation Sequencing), single-channel (SC) and dual-channel (DC) microarrays), have been studied for differential expression and results compared to previous analyses focusing on more restrictive samples. Further, a cluster analysis is presented, to identify the structure of the gene space in the different datasets.

## Methods

This section describes the methodology used for the analysis of the three datasets. The scripts used can be found as supplementary material to this paper (Scripts S1).

**Figure 2:**
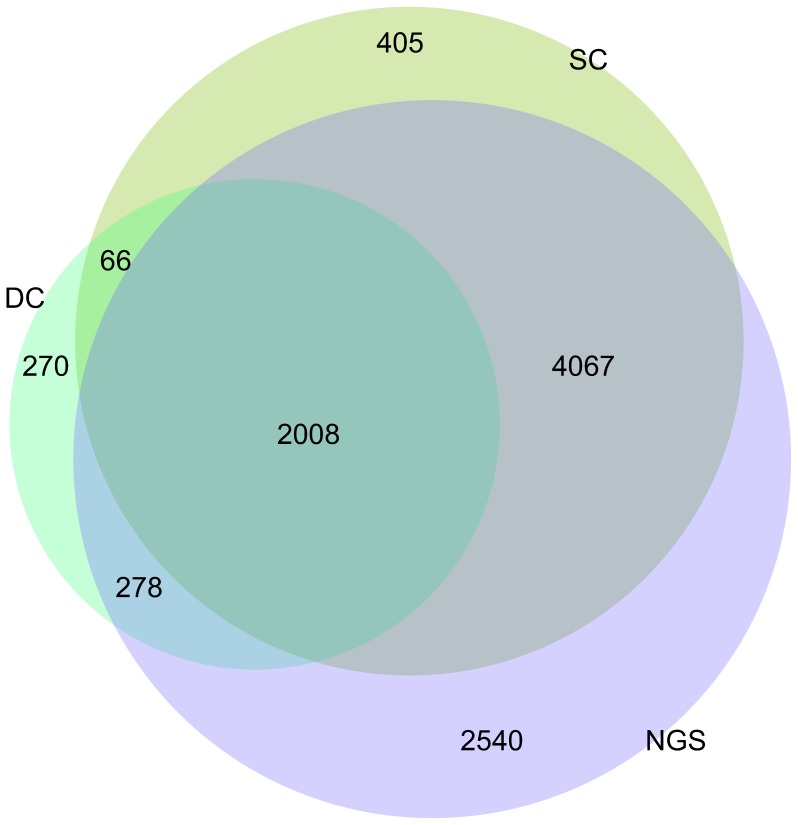
Differentially expressed genes for 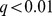. The NGS (Next/Second Generation Sequencing) and SC (Single-Channel) datasets display the largest commonality, while the DC (Dual-Channel) and SC the smallest.

### Datasets and Pre-processing

Three publicly available raw datasets have been downloaded from online resources and used for the analysis. These consist of time series measurements of the fruit fly (*Drosophila melanogaster*) embryo development, and have been measured on three different platforms: single-channel Affymetrix microarrays (referred to as *SC* dataset, Berkeley Drosophila Genome Project [17]), dual-channel microarrays (*DC* dataset, NCBI Gene Expression Omnibus, accession number GSE14086 [18]) and RNA-seq (*NGS* dataset, NCBI Sequence Read Archive, accession number SRP001065 [19]). Table 1 summarises features of these data. The raw data have been pre-processed for differential expression analysis, as follows.

**Figure 3:**
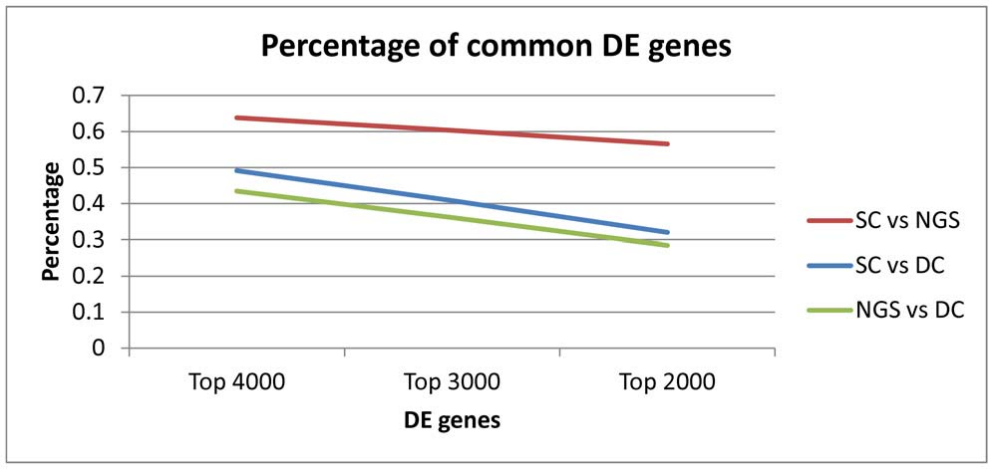
Common differentially expressed genes between dataset pairs with different rank thresholds. For each pair of datasets, only the genes that exist in both datasets are considered. The NGS and SC datasets display the largest commonality, maintained over 50% even for the most restricted DE sets, while the DC and NGS the smallest. However, percentages decrease instead of increasing when restricting the set of genes.

#### Sequencing data

The Illumina Genome Analyzer II reads were mapped to the April 2006 assembly of the *Drosophila melanogaster* genome (dm3, BDGP Release 5) using Tophat (v 1.0.14). This tool also makes use of gene annotations to detect reads that map across known and putative splice junctions. Release 5.12 annotations (Oct. 2008), provided by ‘Flybase’, was downloaded from the UCSC Genome Bioinformatics website. Default Tophat settings were used for mapping.

**Figure 4:**
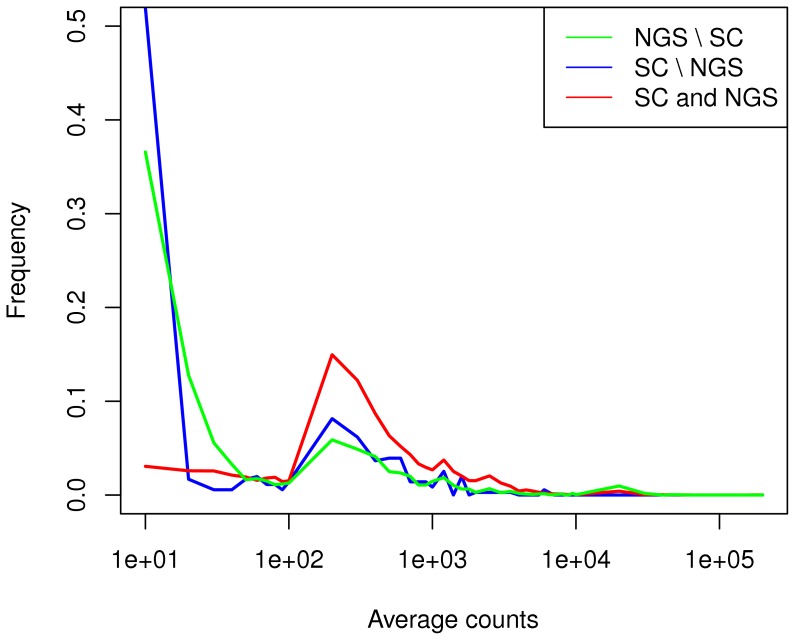
Histogram showing the distribution of average count values (from the NGS dataset) for genes commonly DE in the NGS and SC datasets (6075 genes), versus those DE only in one dataset (2805 for NGS and 356 for SC). Only genes probed on both platforms were considered for this analysis. Uncommon genes display lower counts compared to common. The NGS dataset also identifies a few genes with very large counts.

**Figure 5:**
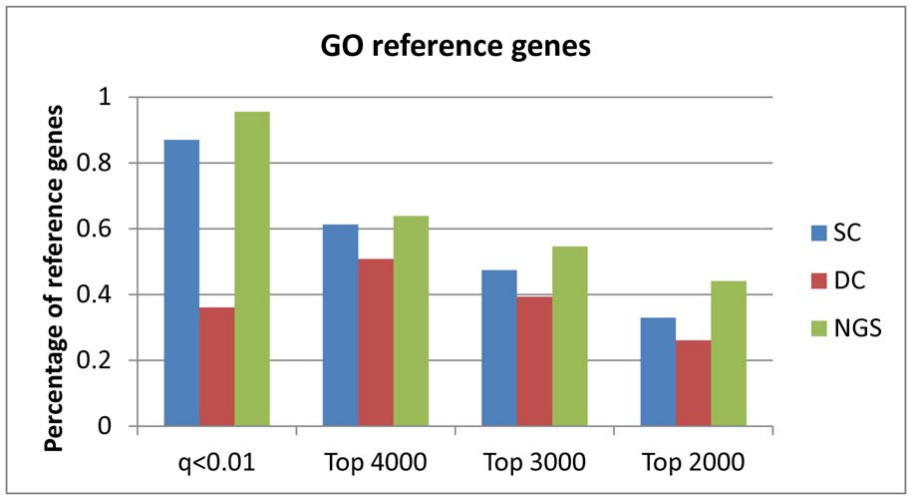
Percentage of reference genes represented in the DE sets obtained from the three datasets. The NGS dataset identifies the largest number of reference genes, and the DC dataset the lowest.

HTSeq, a Python package that provides infrastructure to process data from high throughput sequencing experiments, was used to “count” the number of reads mapping to each gene. Read counts per gene was calculated to be the total number of reads, which mapped uniquely to annotated regions (Release 5.12 annotations). Reads that mapped to more than one location were considered ambiguous and not used. Unique reads, which mapped to a locus with more than one annotated gene, were considered ambiguous and not used (Figure 1).

**Figure 6:**
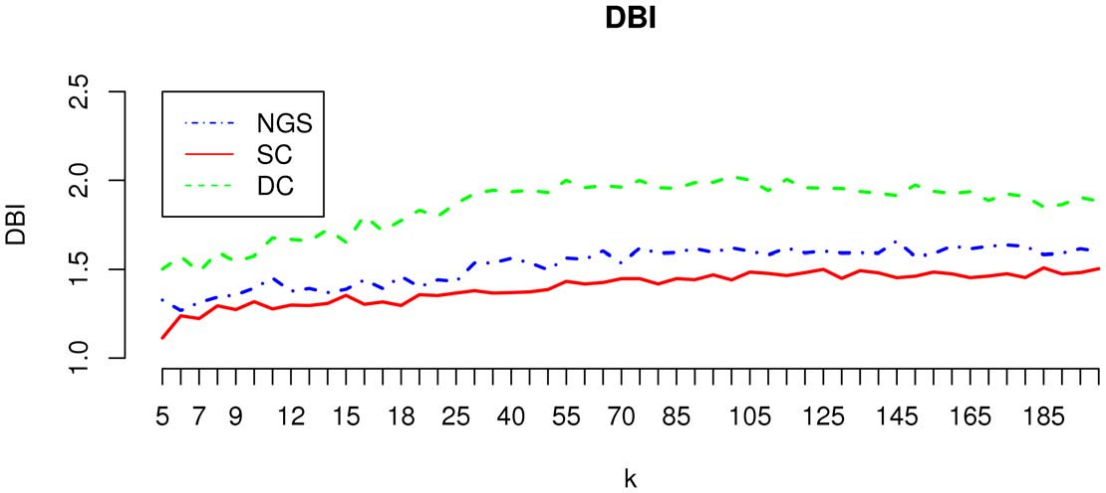
K-means clustering evaluation. Graphs displays DBI values obtained for the three datasets with different 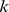.

Reads per kilo base per million reads mapped (RPKM) values were calculated to be used for cluster analysis. Gene length was defined to be the region that encompasses the union of all isoforms of a gene, which do not overlap other genes (Figure 1). RPKM values were log normalised, in order to remove the amplitude/variance dependence in the data (as for microarray normalisation). Clustering of RPKM values without taking logarithms was also performed; but results differed significantly from those for microarrays, with a hierarchical structure imposed on the gene space, due to amplitude differences, so this approach was not pursued here.

**Figure 7:**
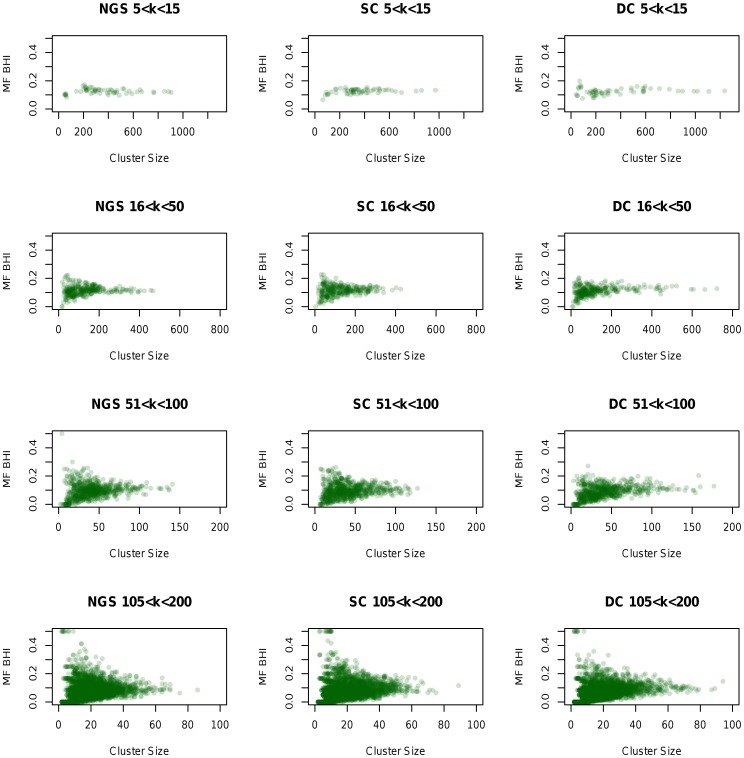
Cluster size and BHI values for different 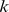. The colour intensity of the spots indicates the number of points falling in the specific area. The graphs show that the gene space is similar for the three datasets. For small 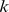, clusters do not have a large BHI, which changes with increasing 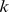, as more clusters become relevantly differentiated.

#### Microarray data

For the two microarray datasets, R software (specifically the *Limma* package [20]) was used for normalisation and expression value extraction. Background subtraction, within-array and between-array normalisation was performed for the DC dataset using the *normexp* and *loess* methods in *Limma*, while for the SC dataset, the RMA method was employed. The resulting normalised datasets were used for differential expression and cluster analysis.

**Figure 8:**
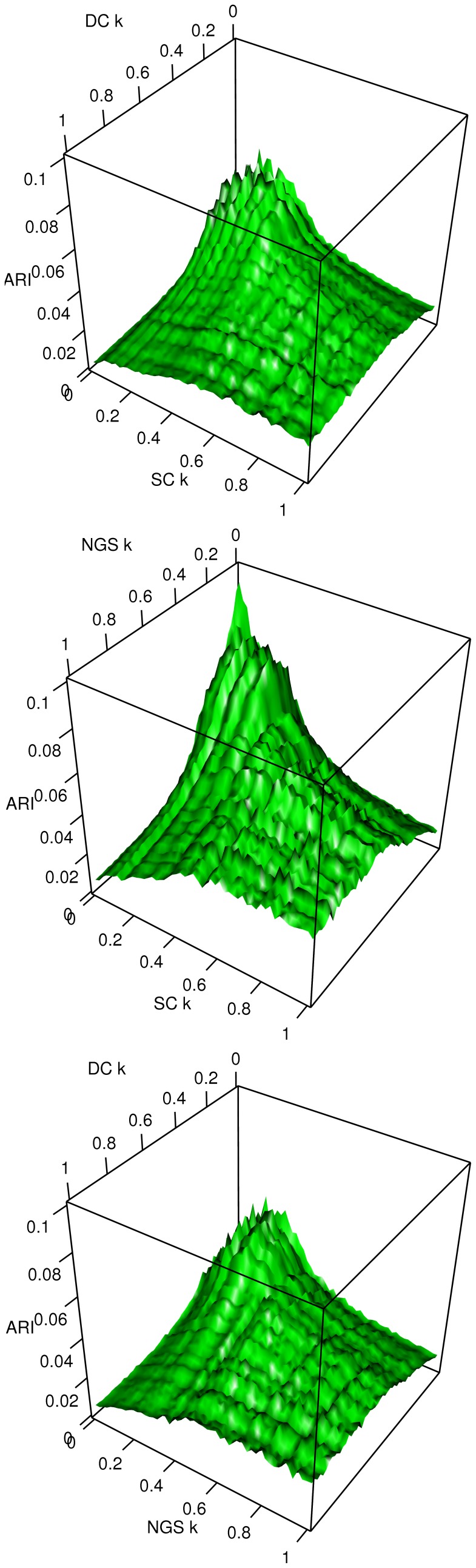
Cluster comparison for dataset pairs. The Adjusted Rand Index (ARI) is displayed for each dataset pair for all combinations of 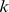 (top to bottom: DC vs SC, NGS vs SC, DC vs NGS). The clusters obtained from SC and NGS are more similar than when comparing the DC dataset with the other two.

### Differential Expression

Differential expression analysis was performed using R software, i.e. the *Limma* package (*lmFit* and *eBayes* methods [21]), for the two microarray datasets (SC and DC), and the *DESeq* package [22] for the sequencing dataset (NGS). For each dataset, we retrieved the set of differentially expressed (DE) genes for at least one time point, compared to the initial one. Given that the data were sampled at different time points and sampling intervals in the three datasets, only those *common to all datasets* were used, resulting in a total of 4 experiments for each. This excluded 42% of the time points from the DC dataset, and 66% from the other two, which was not ideal. Nevertheless, the purpose of the current exercise was to find a ‘kernel’ comparison base, for which to examine all three methodologies, and this is the basis for proceeding with the truncated datasets. As more data become available, relative performance may be assessed on more extensive and complete datasets. (NOTE: in the NGS dataset, genes with null counts in all time points (11% of genes) were removed before differential expression analysis.).

**Figure 9:**
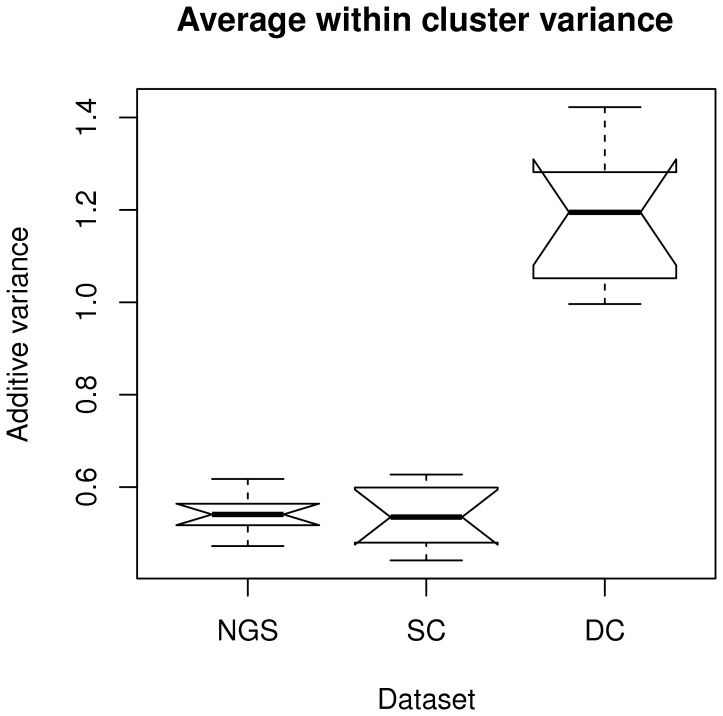
Bicluster average additive variance distribution over ten runs.

**Figure 10:**
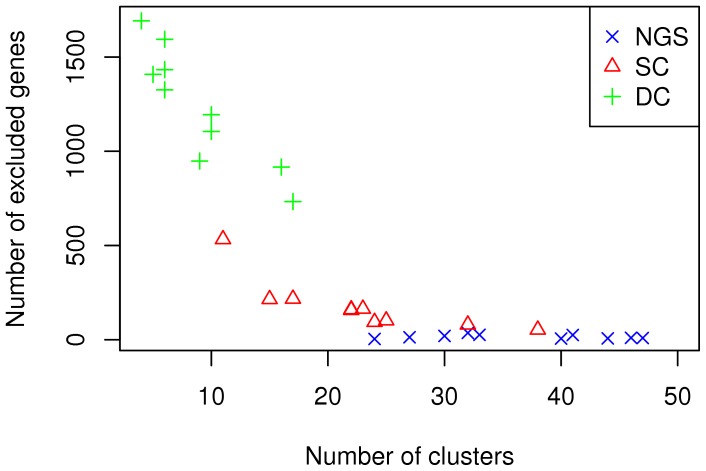
Number of genes not included in biclusters and number of biclusters obtained.

The DE tests employed assume a linear model for the gene expression levels in the two microarray datasets and a negative binomial distribution for counts in the NGS dataset. Based on data replicates, estimates of the expected mean and variance were obtained. The differential expression test between two samples is based on the null hypothesis that the expression values of a gene in both come from the same distribution, with 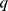-values (adjusted 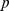-values) obtained for each gene and sample pair ([21], [22] for more detail).

**Figure 11:**
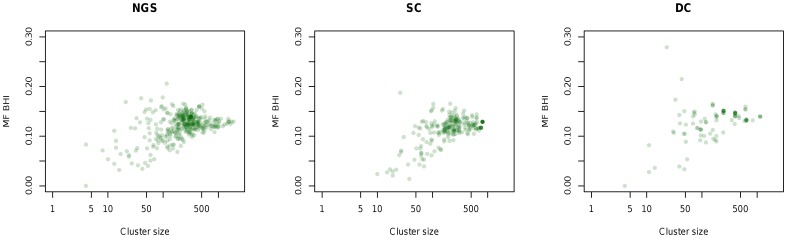
MF BHI and cluster size for biclusters obtained in 10 runs.

The DE sets of genes corresponding to a 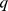-threshold of 0.01 were retrieved and compared for the three datasets. Common and uncommon genes were identified and properties studied. Due to the fact that these sets were very different in size, further analysis of DE sets was performed by selecting the top 4000, 3000 and 2000 genes as ranked by 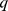-values in each dataset. This enabled analysis of overlapping features of the three datasets, at different granularity levels, without bias from the individual DE null distributions. Firstly, DE genes common to all datasets were studied. Given that some genes were not present in all datasets (as microarray probes differ between platforms, with some having missing values), these were removed from the analysis before each *pair-wise* comparison. Thus, when comparing datasets DC and SC, genes present in both datasets only were considered, whether or not present in dataset NGS. This resulted in eliminating the additional DE genes from the first dataset that were not sampled in the second, to remove bias due to platform sampling range. On average, 70% of genes were retained between the DC and the other two datasets, while about 80% were retained for the NGS vs. SC analysis. While the full data might reasonably be expected to provide additional insight on the extended gene set by platform, truncation was required for the current study with the aim of identifying strictly overlapping data structures for eventual integration, rather than to provide a ranking of technologies.

As indicated in Table 1, each of the three datasets contains at least three replicates for each time point. The NGS replicates are technical (i.e. obtained by sequencing the same sample many times), while those from microarrays are biological (i.e. obtained from different samples). Given that technical replicates differ only in experimental setting (biological diversity is not present), the number of differentially expressed genes in the NGS dataset may be inflated, due to variance underestimation. However, using a pooled approach [22] for variance estimation for these data resulted in a very low number of differentially expressed genes. On investigating the coefficient of variation for replicates in all datasets, larger values were obtained for the NGS dataset. This indicates that these technical replicates are not, in fact, very similar. In consequence, the non-pooled approach was adopted, although this may result in an increase of the number of DE genes retrieved. However, this is expected to have a smaller influence on the top ranked DE genes, which is a further reason to compare these across datasets, and not only the DE sets determined by 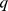-value thresholds (as described above). From the literature, it appears clear that, due to costs involved, RNA-seq experiments are currently performed mostly with technical replicates, e.g. [9], [10], [13], [14], while biological replicates are standard for microarrays [14], [17], [18]. This is due to large costs for library preparation in the case of RNA-seq [23], resulting in a very large fraction of published experiments having only technical or no replicates [24]. Even though replicate type may influence results, analysis of heterogeneous sets for overlapping features is relevant to assessing comparative state-of-the art of the technologies, in the context of recent increased interest in the potential for data integration from different platforms.

### Clustering

To analyse the structure of the gene space in the three datasets, clustering was applied to genes in the top 4000 as ranked by DE analysis on the NGS dataset. Due to platform differences, only 2941 of these genes were measured on all platforms, so these were used for clustering. This approach was selected in order to analyse how the same subset of genes is distributed across the space for the different datasets. Expression values for all time points available in the datasets were used for clustering, i.e. values resulting from *Limma* normalisation for microarrays, and log RPKM [4] values for NGS data. Two clustering algorithms (provided by R software) were employed: K-means with Euclidean distance, and biclustering using the Plaid algorithm; packages *flexclust* [25] and *biclust* [26] respectively. K-means was applied with the preset number of clusters ranging from 5 to 200 (with a step of 1 between 5 and 20 and a step of 5 between 20 and 200). This large range was chosen to explore the structure of the gene space at different granularity levels. The Plaid algorithm was applied 10 times for each dataset. The three datasets were standardised by experiment (i.e. converted to standard scores) prior to clustering, to remove biases related to scale, that may differ from one time point to another, due to experimental differences.

To evaluate clusters obtained from each dataset, several criteria were used. For K-means, the Davies-Bouldin index (DBI) [27] was computed for each run (with a different number of preset clusters), as this indicates whether clusters are both well-defined and well-separated (a lower DBI value indicates *compact* and *distinct* clusters). For biclusters, the within-cluster variance was computed, using the *biclust* package. This gives an indication of the bicluster compactness, with lower variance corresponding to tighter groupings. Additionally, for both K-means and biclusters, the Biological Homogeneity Index (BHI) [28], based on Gene Ontology [29] annotations for molecular function (MF), was computed for all clusters (using package *clValid* [30]). The BHI represents the *percentage of gene pairs* in a cluster with *common annotation*, and allows for evaluation of cluster quality from the biological point of view, complementing the other evaluation criteria based on expression value distance measures alone. Additionally, clusters were compared between datasets using the Adjusted Rand Index (ARI) [31]. This computes a measure of cluster similarity, ranging between 0 and 1, with 0 corresponding to similarity expected from random clusters and 1 to identical clusters.

## Results and Discussion

### Differential Expression

In the first analysis performed, we studied the DE sets of genes obtained from different datasets with 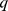-value under 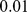. Ideally, the gene sets should show significant overlap, and should be similar in size; in reality, this depends both on the biological variability, measurement parameters and on the DE test, so gene sets vary from one dataset to another. Our aim here is to study the extent of overlap between the three cases. Figure 2 shows the number of differentially expressed genes in each dataset, and overlapping areas. The NGS dataset identified the largest number of genes, in agreement with previous study findings, followed by SC and DC. Datasets SC and NGS show greatest similarity for the DE sets obtained, with a large number of (mostly common) DE genes involved. Compared to this, the DC dataset captures only a restricted DE gene set, implying that the NGS and SC datasets are more sensitive to the DE test. One possible explanation for this may be cross-hybridisation that has been found to decrease the number of DE calls in cDNA microarrays, compared to Affymetrix [32]. The large number of DE genes in the NGS dataset may also be partly due to use of technical replicates; nevertheless, the SC dataset analysis (with biological replicates) also retrieved many DE genes (common). The different samples measure the same process (i.e. embryo development) at the same time intervals, so the genes involved should be the same. Hence, the results suggest that the variance estimation assumption for the technical replicates is reasonably robust. The fact that findings for the NGS dataset are in good agreement with those for the SC dataset also indicates good potential for microarray and RNA-seq data integration in future analysis. Similarity between RNA-seq and the Affymetrix platform has been identified also in previous studies [15].

Since DE analysis based on 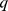-value thresholds is influenced by the DE null distribution of individual datasets, Figure 3 displays the percentage of common genes for dataset pairs, when looking at the top ranked genes for each dataset (i.e. top 4000, 3000 and 2000). The percentage of DE genes common across datasets is expected to increase when the rank threshold decreases, since the more stringent threshold should act as a filter for *non common* genes. Unfortunately, as Figure 3 shows, this is not true for any of the three datasets. This suggests that the DE information on some genes is less precise for *at least one* dataset of the pairing, regardless of thresholds used, probably due to different noise levels and/or other platform differences. This behaviour also occurs when the two microarray platforms are compared, however, so does not necessarily preclude NGS and microarray data integration (not least since dual- and single-channel data have been used in common studies, [33]). It does indicate, however, that reducing noise remains a persistent issue in gene expression analysis, especially technical bias specific to each measurement technology. This requires special attention when dealing with cross-platform integration as it can amplify differences, which can be observed even for one platform due to the intrinsic stochasticity of the gene expression process and normal biological variability.

The genes recorded as differentially expressed in both the NGS and the SC dataset, and those not common to both, were further investigated, and Figure 4 displays the distribution of average count values (number of reads mapping to the specific gene), for differentially expressed genes in the common (6075 genes) and additional categories (2805 for NGS and 356 for SC). The genes specific to only one platform were removed. As the figure shows, in general, a large fraction of uncommon DE genes have very low counts, for both NGS and SC data. This indicates that on low-count transcripts, the two technologies provide complementary information, with more low-count genes identified by the NGS dataset. This can be explained by background noise interference in microarrays hindering correct quantification of rare transcripts. For RNA-Seq this problem does not exist, giving the latter technology an advantage in handling low expression values. The NGS dataset also identifies some highly expressed genes missed by microarray analysis. This might be due to probe saturation in microarrays, not present in RNA-seq. Previous studies have also reported higher DE sensitivity of RNA-seq for large copy-number transcripts (e.g. [14]). However, this property has not been previously identified for low count transcripts also [10], although supported by known characteristics of the different technologies. This might be due to the sequencing depth used in these previous studies [14], which is very important for detecting low count transcripts [6]. For instance, [10] report an average of 11.56 RPKM for their study, while the NGS dataset in this study contains an average of 43.2 RPKM, significantly larger. Of course, it is possible to argue that the low count genes, deemed to be differentially expressed in this dataset, may be an artefact of the use of technical replicates. This has necessitated some further probing of content, and, while not all genes can be used to refute the argument, we have looked at the list of low count (average 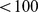) differentially-expressed (DE) genes identified by the NGS dataset, and found examples with strong likelihood of being true positives. A reference set of genes, with high likelihood of being differentially expressed during embryo development, was selected in order to explore whether these were identified from the three datasets, using the methods described. This set of genes consisted of those annotated with the embryo development term in the Gene Ontology database, having ‘gene model status’ with the value ‘Current’ in Flybase (481 genes). Thirteen of these genes are low count genes identified only by the NGS dataset. Additionally, of those low count genes identified by the NGS and not by the SC dataset, 105 are also included in the DC set of DE genes, providing other indication that these are true positives. A further example is gene doublesex, known to have low expression values and to be involved in sex differentiation [6]. This is identified by the NGS dataset as DE and not by the others. These examples indicate that, even if some low count DE genes are artefacts, true positives are found and their identification by the NGS dataset is useful.

Further analysis of the reference gene set is summarised in Figure 5, which shows the proportion of these genes identified from each dataset, together with the different DE sets that apply. Genes that were missing from the three datasets were eliminated before computing these proportions (which are thus based, respectively, on 97, 89 and 98% of the 481 genes actually present in the SC, DC and NGS datasets). This *reduced reference set* of genes is expected to be highly represented in all datasets. In fact, the DC dataset identifies the lowest percentage of reference genes, decreasing further for lower rank thresholds, while the NGS dataset identifies the highest, with over 40% of reference genes present even in the most restricted set, again indicating an advantage over the microarray data. When analysing DE genes identified from the NGS and SC datasets (both low and high count level), 394 reference genes are common to the two DE sets, while 59 and 15 are specific to each (NGS and SC, respectively). This confirms that the two datasets provide complementary DE information, while displaying a large overlap at the same time.

### Clustering of Differentially Expressed Genes

Two clustering algorithms were applied to 2941 genes ranked in the top 4000 by the NGS dataset, common to all three platforms. Clusters are expected to be well-defined and well-separated (i.e. to have small DBI and variance), and display good overlap (large ARI). BHI scores should increase when a larger number of clusters is obtained, as those DE genes included in the same cluster, under these conditions, should share similar processes or function. The rest of this section describes scores obtained for two alternative clustering methods, in order to investigate this hypothesis, and provide insight on the data structure for the three datasets.

#### K-Means clustering

A first analysis of K-Means cluster quality studied numerical separability of groupings obtained. The DBI (Davies-Bouldin Index) values for clusters obtained with the number of clusters (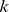) ranging from 5 to 200 are displayed in Figure 6. This indicates better separability for the NGS and SC datasets, compared to the DC dataset, with best values for SC. This shows that the gene space is more structured for these two datasets. At the same time, the figure shows that for all three cases, cluster quality decreases with 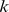, which means that large clusters are better defined than small ones.

For a better view over the data space, the size of clusters and the biological relevance of groups obtained (Molecular Function Biological Homogeneity Index - MF BHI) was also analysed, and is displayed in Figure 7 for different 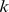 values. In general the gene space structure appears similar for the three datasets. For few predefined centroids (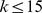), cluster BHI is low, while cluster size range is wide. Increasing 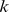, small clusters with larger BHI are differentiated for all three datasets, while large clusters become smaller. This indicates a compact gene space structure, where small relevant clusters become visible only when 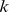 is increased, otherwise cluster relevance remains low. It is important to note that BHI values for all datasets rarely exceed 0.3, which indicates only *moderate biological homogeneity* of clusters. However, this is also found for previous K-means clustering analyses for wild-type gene expression data (e.g. [28], [34]). A similar analysis has been also performed with RPKM values instead of log normalised; however, the approach is less stable for NGS compared to microarrays, for small 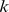 - as noted earlier. Thus, due to large variance for gene expression levels, genes with extreme expression values were clustered together, forming isolated islands around the main gene grouping. In consequence, only the log-normalised values are discussed here.

In order to compare the clustering results from the three datasets, the Adjusted Rand Index (ARI) was computed for dataset pairs, and Figure 8 displays the resulting values for all 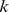. In general ARI values for the SC and NGS dataset are larger than those of the other two pairs, again confirming the similarity between the two datasets. For all three pairs, similarity decreases when 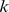 increases, showing that although the cluster-space structure is maintained, cluster content may differ.

#### Plaid

Average within-cluster variance for *biclusters* found over ten runs are displayed in the form of boxplots in Figure 9. This indicates that the SC and NGS datasets display the lowest within-cluster variability, and DC the largest. Given that the Plaid algorithm may not include all genes in clusters, the number of genes not taken into account and the number of clusters identified in each corresponding run are displayed in Figure 10. For the DC dataset the number of clusters is smaller than for the other two, while many genes are not included in clusters. This, together with the higher variance, indicates less separability for this dataset, as shown also by DBI values presented earlier for K-Means analysis.

Additionally, Figure 11 displays cluster size and MF BHI values for the biclusters. BHI values are modest for all three datasets, with the highest obtained for moderate cluster sizes, and no significant differences detectable between the three cases. This shows that even for the datasets that apparently have better separability and tighter clusters (SC and NGS), biological relevance of clusters is reduced when analysing gene expression levels. However, similar to the K-Means analysis, these results are in agreement with other studies focusing on BHI values for clustering gene expression data ([28]).

### Conclusions

An analysis of three types of gene expression data for *Drosophila melanogaster* embryo development time series was presented; these include both dual- and single-channel microarrays, and RNA-seq. The aim was to identify similar and complementary features of these datasets, with a view to investigating the potential for future data integration from the three platforms. For this, some truncation of the datasets was needed (genes common to the different platforms, common time points). Although the data eliminated provide further information on the process studied, truncation was required in order to obtain a common comparison base. As more datasets become available, a more extensive analysis of the different criteria can be performed. A sensitivity analysis was employed to study the sets of differentially expressed (DE) genes obtained with 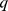-values under 0.01 and different rank thresholds, and, subsequently, to assess cluster quality on applying two clustering algorithms: Euclidean K-means and Plaid biclustering.

Differential analysis indicated, in agreement with the literature, that the NGS and SC datasets are more sensitive to the DE test, with large numbers of DE genes identified, in contrast to findings for the DC dataset. Although the three datasets contain different samples, they measure the same process of embryo development, so they should identify similar DE genes. However, agreement on which genes are DE between the three datasets is not complete, even when looking at top ranked genes. The highest commonality is found between the NGS and SC datasets, with lowest between NGS and DC. These findings are in agreement with previous studies of differential expression (e.g. [14]), although those have been performed in a less broad setting, i.e. by using the same sample for all experiments. This suggests that integration of highly heterogeneous datasets may be feasible. Many of the uncommon DE genes (NGS vs SC dataset) have relatively low expression values, with a larger number of such genes identified by RNA-seq data. Additionally, some very abundant genes have been identified only by RNA-seq data. This suggests that, as postulated, the new technology has an advantage in quantifying extreme transcription levels.

K-Means clustering indicated similarities between the structure of the data space for the three datasets, with less separability for the DC data compared to the other two. However, BHI values showed clusters to be comparable, in terms of biological relevance, for all three datasets. When cluster assignment was compared, the SC and NGS datasets showed smallest differences (larger ARI). Clustering with the Plaid algorithm confirmed the similarities between the SC and NGS datasets and the better separability of clusters.

In conclusion, results suggest that the three datasets provide both overlapping and complementary information on the gene space: for DE analysis, the NGS and SC dataset are mostly similar, but provide complementary information on extreme expression values; for clustering, the NGS dataset appears to display a gene space structure similar to the SC, while the DC data are less separable.

## Supporting Information

Scripts S1 This file contains example R scripts used for the study presented here. The several sections in the file correspond to the different DE and clustering analyses performed.
(TXT)
